# In Vitro Investigation of Biological and Toxic Effects of 4-Octylphenol on Human Cells

**DOI:** 10.3390/ijms252313032

**Published:** 2024-12-04

**Authors:** Antonio Massimiliano Romanelli, Antonio Montefusco, Silvia Sposito, Bernardina Scafuri, Ivana Caputo, Gaetana Paolella

**Affiliations:** 1 Department of Chemistry and Biology, University of Salerno, 84084 Fisciano, Italy; aromanelli@unisa.it (A.M.R.); amontefusco@unisa.it (A.M.); bscafuri@unisa.it (B.S.); gpaolella@unisa.it (G.P.); 2 Department of Medicine, Surgery and Dentistry “Scuola Medica Salernitana”, University of Salerno, 84084 Fisciano, Italy; ssposito@unisa.it; 3 European Laboratory for the Investigation of Food-Induced Diseases (ELFID), University of Salerno, 84084 Fisciano, Italy

**Keywords:** 4-octylphenol, 4-nonylphenol, HepG2 cells, cytotoxicity, unfolded protein response, autophagy, oxidative stress, ADMET prediction

## Abstract

Alkylphenols are byproducts of anthropogenic activities that widely contaminate waters, soils and air; among them, the most represented are 4-nonylphenol (4-NP) and 4-octylphenol (4-OP). These compounds tend to bioaccumulate in animal and plant tissues and also represent a risk to human health. Indeed, humans are constantly exposed to alkylphenols through ingestion of contaminated water and food, inhalation and dermal absorption. In the present work, we characterized the cytotoxic ability of 4-OP towards several human cell lines, representing the potential main targets in the human body, also comparing its effect with that of 4-NP and of a mixture of both 4-OP and 4-NP in a range of concentrations between 1 and 100 μM. Viability assays demonstrated that each cell type had a peculiar sensitivity to 4-OP and that, in some cases, a combination of the two alkylphenols displayed a higher cytotoxic activity with respect to the single compound. Then, we focused our attention on a liver cell line (HepG2) in which we observed that 4-OP increased cell death and also caused interference with protective physiological cell processes, such as the unfolded protein response, autophagy and the antioxidant response. Finally, our experimental data were compared and correlated with ADMET properties originating from an in silico analysis. Altogether, our findings highlight a possible contribution of this pollutant to deregulation of the normal homeostasis in human liver cells.

## 1. Introduction

Environmental pollution originating from anthropogenic activities is one of the major problems plaguing modern society. Many byproducts of industrial processes are released into the environment and enter the food chain, undergoing biomagnification phenomena and representing a serious risk to human health. Among recent emerging pollutants, alkylphenols ethoxylates (APEOs) are a group of non-ionic surfactants used in several industrial applications, such as paper and textile production, pesticides, cosmetics and detergent formulations. The most notable members of APEOs are nonylphenol ethoxylate and octylphenol ethoxylate, which represent approximately 80% and 20% of the APEO production [1]. APEO release into the environment can occur during their synthesis, incorporation into finished products or during the disposal phase [1]. When they reach wastewater treatment plants, they undergo biodegradation processes mediated by microorganisms, with a gradual shortening of the ethoxyl chain [2] and the production of two main alkylphenols (APs): 4-nonylphenol (4-NP) and 4-octylphenol (4-OP). APs are found in wastewater in highly variable concentrations depending on the industrialization of the area and on the efficiency of the wastewater treatment plants [1]. They have also been found in different environmental matrices, such as groundwater, sediments, surface waters, soils and air [2]. Consequently, a certain degree of contamination by APs has often been detected in food (including products for infants) and drinking water [2,3]. Consistent restrictions about APEOs and AP use and emission occur in Western Countries, however, there is not a sufficiently restricted regulation in Asian and South American Countries [2].

The wide environmental diffusion of APs represents a serious ecotoxicological risk for aquatic ecosystems. In particular, since some isomers of 4-NP and 4-OP, especially those branched, mimic the structure of the hormone 17-β-estradiol, they are able to compete with it for binding to the receptor. Therefore, these compounds can be considered endocrine-disrupting chemicals (EDCs) with deleterious effects on the reproduction and development of aquatic organisms [2,4,5]. Through different routes, including the ingestion of water and contaminated food, inhalation and dermal absorption, humans are constantly exposed to APs [6,7], which, owing to their properties of solubility and hydrophobicity, tend to bioaccumulate in tissues and body fluids. 4-NP and 4-OP have been found in maternal blood plasma and amniotic fluid, indicating a risk of prenatal exposure to these pollutants with possible consequences for fetal development [8]. The presence of 4-NP and 4-OP has also been recorded in adipose tissue [9], breast milk [10] and urine samples [11]. To date, the majority of the scientific literature regarding AP toxicity deals with 4-NP [1,5]; however, evidence of the toxic effects of 4-OP and its interference with physiological processes is increasing. The negative effects of 4-OP on the reproductive system can lead to embryo implantation failure, spontaneous abortion and preeclampsia [12,13]. Other observations regard the cytotoxic actions on the male reproductive system [14]. 4-OP can also induce neuronal damage and neurobehavioral abnormalities [15,16]. Bianco et al. [17] showed that there was an accumulation of 4-OP in different brain areas of treated rats, suggesting that lipophilic EDCs can cross the blood–brain barrier. The chronic exposure to 4-OP also seemed to alter hepatic and fat metabolism in rats [18,19], thus increasing the possible development of metabolic-related diseases. The immune system is also affected by exposure to APs, as demonstrated, for example, by the aggravation of symptoms of atopic dermatitis in rats exposed to 4-OP through a Th2-mediated immune response [20]. Finally, based on still limited experimental data and observations, a contribution of 4-OP to epithelial–mesenchymal transition and tumor progression, in particular in hormone-responsive tissues, has been suggested [21].

With the aim of expanding the knowledge on the cytotoxic potential of 4-OP, we investigated the effect of the most diffused 4-OP isomer, i.e., the branched 4-tert-OP (here simply named 4-OP), on different human cell lines (hepatic, intestinal, pulmonary, epidermal, renal) representing those cell types potentially more exposed to this contaminant. We were interested in highlighting the differences in the sensitivity of each cell type to 4-OP and also to compare the cytotoxicity of 4-OP with that of 4-NP and a mixture of both, looking for eventual synergic effects of their combination. We also compared our experimental data with ADMET (Absorption, Distribution, Metabolism, Excretion, and Toxicity) properties predicted in silico. We further focused our attention on the potential damaging effects of 4-OP in a model of human liver cells (HepG2 cell line), as the liver is one of the first lines of defense against xenobiotics, which in some cases may alter organ functions [22]. In this hepatic in vitro model, we demonstrated that 4-OP caused an increased cell death, as well as interference with protective cell processes, such as the unfolded protein response (UPR), autophagy and the antioxidant response.

## 2. Results

### 2.1. Effects of 4-OP on Viability of Human Cell Lines

**Figure 1:**
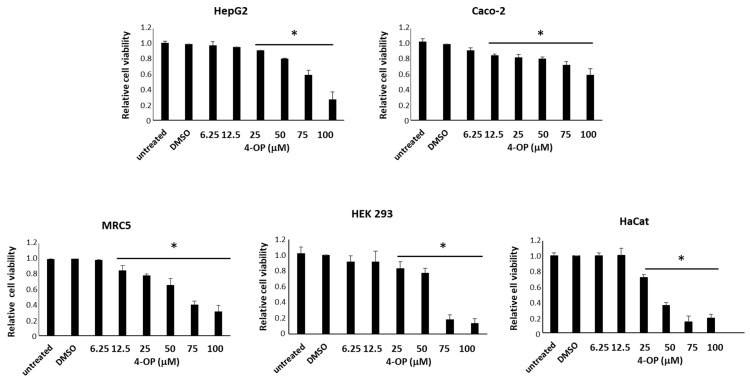
Cell viability assay on different human cell lines treated for 24 h with 4-OP in the range of concentrations from 6.25 μM to 100 μM. Values are the means ± standard error (SE) of three independent experiments performed in triplicate. Statistical analysis was performed using the Student’s *t*-test. * *p* < 0.05 vs. cells treated with the vehicle (DMSO).

We performed a 3-(4,5-dimethylthiazol-2-yl)-2,5-diphenyltetrazolium bromide (MTT) cell viability assay to evaluate 4-OP cytotoxicity after exposure to different concentrations of the pollutant for 24 h. We employed five different cell lines representative of the potential main targets of environmental contamination by APs. We observed that 4-OP caused a reduction in cell viability in all cell lines tested; such a reduction was dose-dependent but with a peculiar trend in each cell line, indicating a different sensitivity to the pollutant (Figure 1). Data from MTT assays were used to calculate the IC_50_ for 4-OP (Table 1, first line), which highlighted that HepG2 and Caco-2 cells (hepatic and intestinal cells, respectively) were the less responsive ones compared to the others, even if HepG2 cells were more sensitive to high 4-OP concentrations than Caco-2 cells. MRC5 and HEK cells (pulmonary and renal cells, respectively), showed an intermediate sensitivity, whereas HaCat cells (dermal keratinocytes) showed the highest sensitivity to 4-OP exposure.

**Figure 2:**
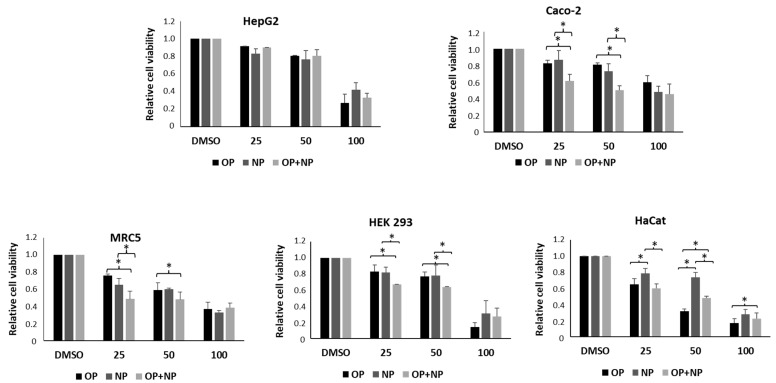
Comparison of the effect on cell viability of 4-OP with the effect of 4-NP and with the effect of a mixture of them (each used at half the concentration of the pure compound) in the range of concentrations from 25 μM to 100 μM. Values are the means ± SE of three independent experiments performed in triplicate. Statistical analysis was performed using the Student’s *t*-test. * *p* < 0.05 as indicated.

Next, we compared the effect of 4-OP and 4-NP in each cell line and also focused on the cytotoxic effect of their combination (Figure 2 and Table 1). In HepG2 cells, we did not find significative differences in the effect of 4-OP, 4-NP and their combination, at all concentrations tested; thus, calculated IC_50_ values were very similar. In Caco-2 cells, 4-OP and 4-NP exerted a similar effect at all concentrations evaluated, even if a suggestion of a higher sensitivity to 4-NP came from a slightly lower IC_50_ with respect to the IC_50_ for 4-OP. In addition, their mixture produced a synergistic effect at 25 and 50 μM, as also confirmed by a significantly lower IC_50_ of the mixture compared to the IC_50_ of each single AP. In MRC5 cells, no significative differences in the effect of 4-OP and 4-NP were observed at all concentrations tested, even if there was a slightly lower IC_50_ for 4-OP than for 4-NP; also, in this case, the mixture caused a synergistic effect clearly evident at 25 μM, which was confirmed by the very low IC_50_ of the mixture compared to the IC_50_ of each single AP. In HEK 293 cells, we did not register significative differences in the effect of 4-OP and 4-NP at all concentrations tested; but, again, we observed a synergistic effect of the mixture at 25 and 50 μM; however, this synergy was not highlighted by IC_50_ values, which were very similar. Finally, HaCat cells clearly showed a higher sensitivity to 4-OP than to 4-NP, in particular at 25 and 50 μM, as also confirmed by respective IC_50_ values; consequently, the mixture seemed to attenuate the cytotoxic effect of 4-OP.

### 2.2. Effects of 4-OP on Cell Cycle

**Figure 3:**
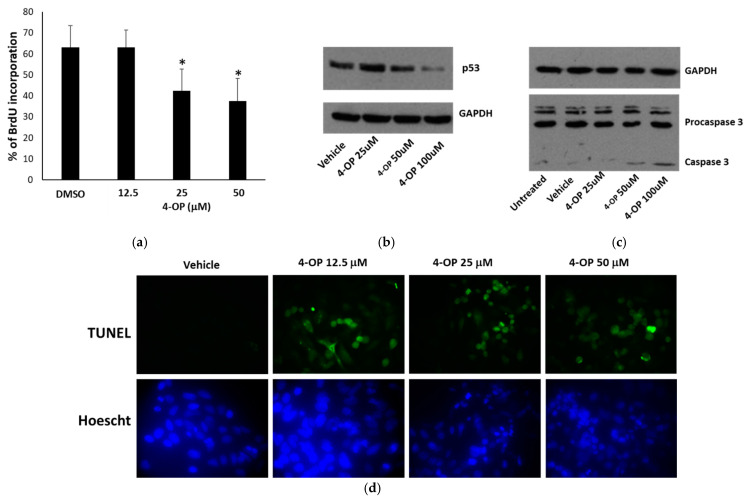
Effect of 4-OP on proliferation and apoptosis in HepG2 cells. (**a**) BrdU incorporation in cells treated for 24 h with 25 and 50 μM of 4-OP. Data are reported as the mean ± SE from three independent experiments. * *p* < 0.05 versus vehicle-treated cells (DMSO). (**b**) Western blot of p53 in cells treated for 24 h with 4-OP. (**c**) Western blot of procaspase and cleaved caspase-3 in cells treated for 8 h with 4-OP. In (**b**,**c**), GAPDH is reported as the internal reference. (**d**) TUNEL assay in cells treated for 24 h. Apoptotic nuclei are in green, and total Hoechst-stained nuclei are in blue. Magnification 40×, with oil.

We further investigated the biological effects of 4-OP focusing our attention mainly on the HepG2 cell line. Considering the cytotoxicity observed in MTT assays, we investigated whether 4-OP was able to affect cell proliferation or cell death or both. We analyzed cell cycle progression in the presence of 4-OP by performing a bromodeoxyuridine (BrdU) incorporation assay and observed that 4-OP reduced the entry into the S-phase at concentrations of 25 and 50 μM (Figure 3a). Given the importance of the p53 protein in controlling cell proliferation [23], we evaluated its expression in the presence of 4-OP. Western blot analysis highlighted that p53 expression was decreased at higher concentrations used (Figure 3b); this result was compatible with a reduction in proliferation. We also studied the occurrence of apoptosis, by observing the appearance of cleaved caspase 3 via Western blotting and chromatin fragmentation by performing a Terminal deoxynucleotidyl transferase dUTP nick end labeling (TUNEL) assay. Both approaches revealed that 4-OP was able to induce apoptosis (Figure 3c,d).

### 2.3. Effects of 4-OP on UPR

**Figure 4:**
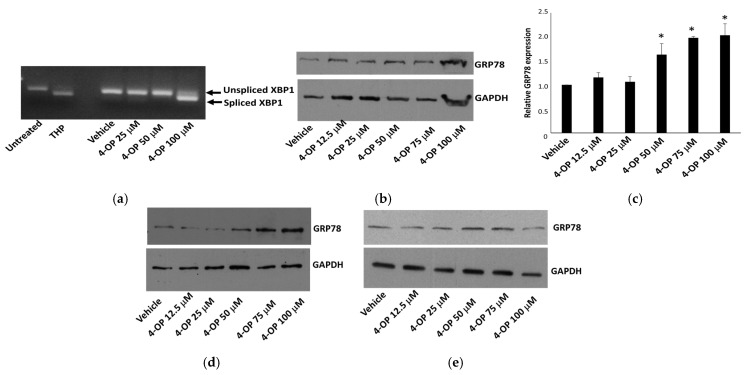
Effect of 4-OP on UPR and ER stress. (**a**) Visualization on a 2.5% agarose gel of PCR products revealing unspliced and spliced forms of XBP1 in HepG2 cells treated for 4 h. THP 1 µM represents the positive control. (**b**,**c**) Western blot and densitometric analysis, respectively, of GRP78 in HepG2 cells treated for 24 h with 4-OP. Data are reported as the mean ± SE from at least three independent experiments. * *p* < 0.05 versus vehicle-treated cells. (**d**,**e**) Western blot of GRP78 in MRC5 and Caco-2 cells, respectively, treated with 4-OP for 24 h.

To evaluate whether the exposure to 4-OP could induce the UPR indicating a condition of ER-stress, we analyzed two typical early markers of this process. First, we monitored, via a conventional PCR, the appearance of the X-box binding protein 1 (XBP1) spliced form in HepG2 cells treated for 4 h with different amounts of 4-OP. In our experimental condition, splicing was clear at 100 μM of 4-OP (Figure 4a). The ER-stress inducer thapsigargin (THP) was employed as a positive control in these experiments. We also analyzed the expression of the chaperone glucose-regulated protein (GRP)78, which appeared to increase in a dose-dependent manner reaching higher induction at 100 μM of 4-OP (Figure 4b,c). Similarly, GRP78 expression was markedly induced in MRC5 cells (Figure 4d). Instead, the effect was less evident in Caco-2 cells (Figure 4e). On the whole, data suggested that 4-OP was able to trigger the UPR in human cells, as already described for 4-NP [24].

### 2.4. Effects of 4-OP on Autophagy

**Figure 5:**
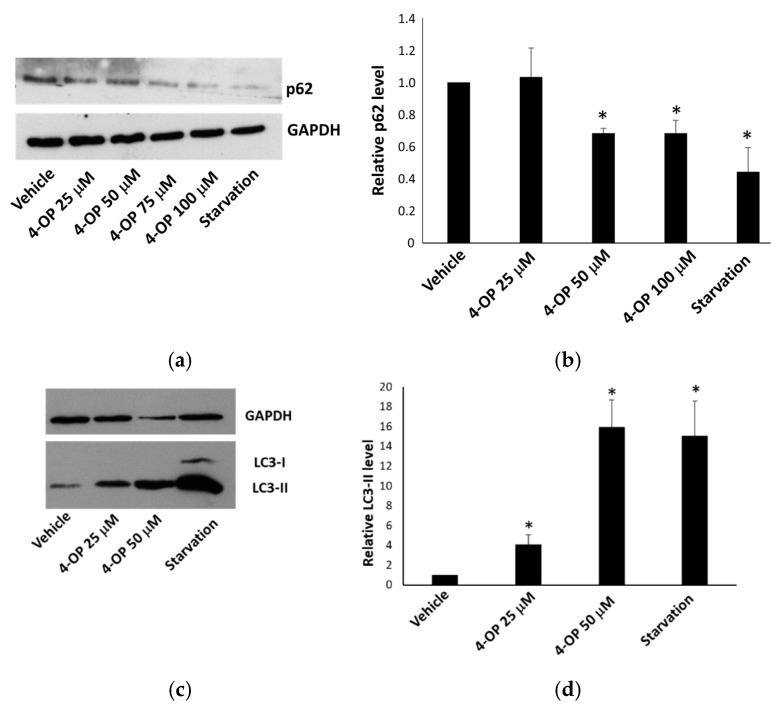
Effect of 4-OP on autophagic markers. (**a**,**b**) Western blot and densitometric analysis, respectively, of p62 in HepG2 cells treated for 4 h with 4-OP. (**c**,**d**) Western blot and densitometric analysis, respectively, of LC3-II in HepG2 cells treated for 24 h with 4-OP. Starvation represents the positive control. Data are reported as the mean ± SE from three independent experiments. * *p* < 0.05 versus vehicle-treated cells.

**Figure 6:**
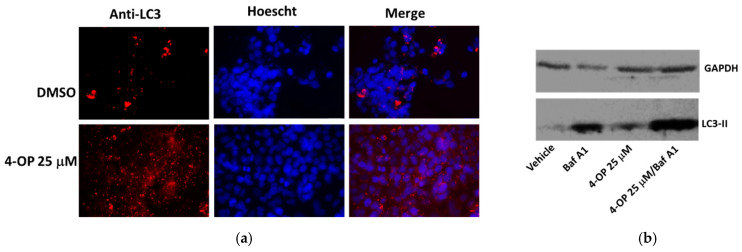
Effect of 4-OP on autophagic markers (**a**) Immunofluorescence images of HepG2 cells treated with 4-OP for 24 h and stained with anti-LC3 antibodies (red); Hoechst-stained nuclei are in blue. Magnification 40×, with oil. (**b**) Western blot showing LC3-II levels in the presence of Baf A1, 4-OP and a combination of both; HepG2 cells were treated for 4 h with 4-OP and then for a further 20 h with Baf A1 at 50 nM.

We investigated the ability of 4-OP to modulate autophagy by analyzing two markers of this process. During the formation of autophagolysosomes, the protein p62 is degraded, whereas the microtubule-associated protein 1A/1B-light chain 3 (LC3)-I is converted to LC3-II. When we monitored the level of these proteins in HepG2 cells, we found that p62 was decreased and LC3-II was increased after treatments with 4-OP for 4 h and 24 h, respectively (Figure 5), thus suggesting that autophagy was stimulated by the compound. Starvation was used as an inducer of the process. In addition, we performed immunofluorescence staining for LC3, highlighting the presence of perinuclear puncta corresponding to autophagosomes (Figure 6a). We observed an accumulation of autophagosomes in cells treated with 4-OP compared with cells treated with vehicle alone. Finally, we used bafilomycin A1 (Baf A1), an inhibitor of the autophagic flux, to confirm that 4-OP was causing an increase in autophagy and not its blockage; indeed, by adding Baf A1 to cells, pre-treated with 4-OP, we observed the further accumulation of LC3-II, indicating that 4-OP was increasing the autophagic flux (Figure 6b). Altogether, data are in line with the idea that 4-OP could modulate autophagy in HepG2 cells.

### 2.5. Effects of 4-OP on Antioxidant Enzymes

**Figure 7:**
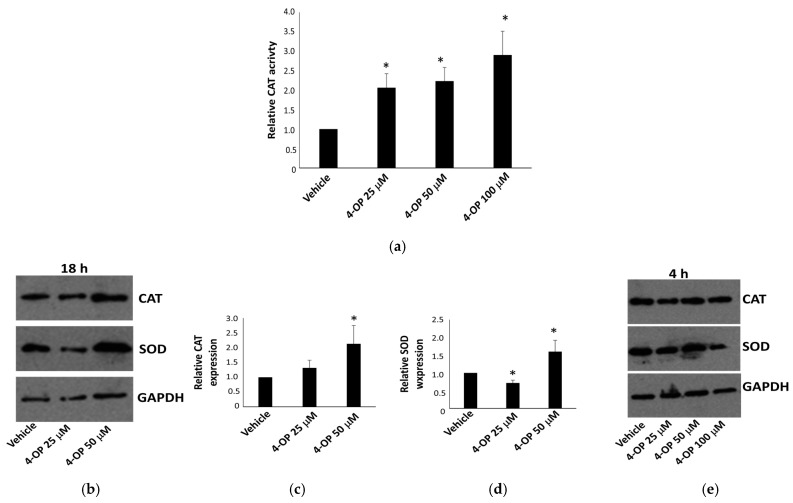
Effect of 4-OP on antioxidant enzymes. (**a**) CAT assay on HepG2 cells after 18 h of treatment with 4-OP. (**b**) Representative Western blot showing CAT ad Sod levels after 18 h of treatment with 4-OP. (**c**,**d**) Densitometric analyses of CAT and SOD levels, respectively, in HepG2 cells treated for 18 h with 4-OP. (**e**) Western blot showing CAT ad SOD levels after 4 h of treatment with 4-OP. Data are reported as the mean ± SE from three independent experiments. * *p* < 0.05 versus vehicle-treated cells.

The effect of 4-OP on the antioxidant system was first evaluated by considering the activity of an important antioxidant enzyme, catalase (CAT). In HepG2 cells, we observed an increase in CAT activity in a dose-dependent manner after treatments for 18 h with 4-OP (Figure 7a). We also evaluated the CAT expression level, and we found that the CAT protein level increased and was significantly more expressed compared to the control at 50 μM of 4-OP (Figure 7b,c). On the other hand, the expression level of another marker of oxidative stress, superoxide dismutase (SOD), appeared to be lower in cells treated with 25 μM of 4-OP than in vehicle-treated cells and increased at the concentration of 50 μM (Figure 7b,d). After only 4 h of treatment, the CAT level did not vary, whereas we observed a reduction in the SOD level at the concentration of 100 μM of 4-OP (Figure 7e). On the whole, data suggested that a slight but significant perturbation of the antioxidant response was occurring in HepG2 cells exposed to 4-OP.

### 2.6. In Silico Prediction of ADMET Properties of 4-OP

The ADMET profile of 4-OP is presented in Table 2. Among the output data obtained using the platform ADMET Lab2, we chose those parameters useful for a comparison with the experimental data. In particular, 4-OP is predicted to be permeable to the epithelial barrier and to be absorbed by the human intestine. Moreover, 4-OP could be found in human plasma and can cross the blood–brain barrier. Metabolism parameters revealed a high activity of 4-OP towards cytochrome P450 isoenzymes. Unexpectedly, no prediction of hepatotoxicity has been reported, whereas a high and moderate degree of skin and respiratory toxicity, respectively, has been predicted. A high probability of 4-OP being an agonist of estrogen receptors also emerged, in line with its endocrine-disrupting activity [4]. Finally, a moderate and a high effect on the heat shock response and membrane mitochondrial potential, respectively, has been predicted.

## 3. Discussion

4-OP belongs to a class of nonionic surfactants, APs, that, for their chemical features, are often used in multiple industrial applications and, once released into the environment, accumulate in waters, sediments and air [1,4]. The main route of exposure for humans is the ingestion of contaminated water and food; therefore, the gastrointestinal system and the liver, which is the main organ involved in detoxifying foreign substances [25], could be particularly compromised by APs. Dermal absorption and inhalation represent two other important routes of exposure; thus, pulmonary and dermal pathological conditions could develop after prolonged exposures. Even if 4-OP is less represented than 4-NP in environmental matrices, its potentially damaging effects on living organisms are not negligible and its action on cells and tissues requires further investigation.

In the present study, we evaluated the cytotoxic effects of 4-OP, for which the effects are less explored than 4-NP, in different human cell types, representing the potential main targets after exposure to pollutants. Interestingly, the ADMET prediction suggested that 4-OP had a high capability of crossing epithelial barriers and being absorbed; 4-OP is also predicted to be consistently present in the human plasma indicating the possibility to reach all human districts, accordingly to experimental data regarding its presence in various human organs and biological fluids [8,9,10,11]. A moderate score regarding the blood–brain barrier penetration is in line with findings about brain accumulation and damage [15,16,17]. Cell viability assays after treatments for 24 h with a wide range of concentrations of 4-OP highlighted that different cell types displayed differential sensitivity to the compound, with dermal keratinocytes (HaCat) being the most sensitive and intestinal epithelial cells (Caco-2) being the most resistant to 4-OP-induced cytotoxicity. In line with these experimental data, the ADMET in silico prediction showed a high score of skin sensitization. Furthermore, from our analysis, it emerged that in four cell lines (HepG2, Caco-2, MRC5, HEK-293), 4-OP-induced effects were similar to those evoked by 4-NP, whereas, in HaCat cells, 4-OP was significantly more cytotoxic than 4-NP at intermediate concentrations (25 and 50 μM). Interestingly, it has recently been demonstrated that precisely in HaCat cells, 4-OP exerted potential skin-sensitization and immunomodulatory effects [26]. We also compared the effects of single APs with a mixture of both. In Caco-2, MRC5 and HEK-293 cells, treatments with the mixture produced a slight but significant synergistic effect, suggesting the possibility that the two APs could act through partially different mechanisms. We did not observe such a synergistic effect in HepG2 cells, nor in HaCat cells; in this last case, the mixture was less effective than 4-OP alone. Our data regarding the mixture agree with studies demonstrating some synergistic effects of EDCs. Gan et al. demonstrated that 4-NP and bisphenol A have synergistic action in reducing the viability of RWPE-1 cells (a human prostate epithelial cell line) [27]. In an in vivo model, the combination of xenoestrogens enhanced the pathological abnormalities of the mouse female reproductive tract [28]. In mouse testes, the steroidogenesis pathway was affected in a more complex way by treatments with a mixture of EDCs (including 4-NP and 4-OP) compared with the corresponding single exposure [29].

Similarly to 4-NP, 4-OP can partially accumulate in the liver and affect liver cell homeostasis [30]. A metabolomics study revealed that 4-OP low-dose exposure disturbed nucleic acid and amino acid metabolism in the mouse liver [31]. In a study on pregnant rats, 4-OP exerted adverse effects on fat metabolism in the liver and adipose tissue [19]. In addition, exposure to 4-OP caused acute hepatotoxicity in immature male rats [29] and several adverse effects on the spleen and liver tissues of adult rats exposed to 4-OP during prenatal life [32]. Unexpectedly, ADMET analysis showed a low degree of human hepatotoxicity; however, high scores relative to interference with the detoxifying activity of cytochrome P450 enzymes, which are typical of the liver [22], have been predicted.

Here, we used HepG2 cells to investigate how 4-OP may interfere with some basal physiological processes in human liver cells. In this regard, HepG2 cells are considered a good in vitro model for toxicological studies [33]. First, we evaluated how 4-OP could modulate cell proliferation and death. We found that 4-OP reduced the entry into S-phase and, in parallel, induced apoptosis in HepG2 cells, as highlighted by the activation of caspase-3 and chromatin fragmentation. These data are in line with those previously reported, which demonstrated how cells responded to the damage induced by 4-OP and other APs, by blocking cell cycle progression and inducing programmed cell death [15,24,34,35,36].

It has often been reported that apoptosis induced by EDCs can be caused by an unsolved ER-stress [24,37]. In Xenopus laevis embryos, ER stress has been described among the biological effects of 4-OP exposure that induced head abnormal development [16]. In addition, prolonged exposure to 4-OP or bisphenol caused ER stress in a mouse model of type 1 diabetes mellitus [38]. In the present work, we investigated the ability of 4-OP to modify two early markers of UPR, the response triggered by ER stress. We found that both the XBP1 spliced form and GRP78 were increased after treatments with 4-OP. GRP78 was also increased in MRC5 and Caco-2 cells, indicating that the occurrence of 4-OP-induced UPR could be a general response in different cell types. These data are also confirmed by the ADMET prediction from which a moderate probability of modulating the heath shock response, including the UPR, emerged. In human cells, a close correlation among ER stress, the activation of apoptosis and Ca^2+^ homeostasis has been demonstrated [39,40]. Consistently with this observation, previous works showed that 4-NP caused the abnormal elevation of intracellular Ca^2+^ levels in different cell models [41,42] and in the pancreas of a mouse model [38]. In line with these observations, we preliminary obtained an indirect demonstration of the ability of 4-OP to mobilize Ca^2+^ ions from intracellular stores, by assaying the activity of a cytosolic Ca^2+^-dependent enzyme (type 2 transglutaminase) in Caco-2 cells incubated in a Ca^2+^-free condition; in this experimental condition, we found that ionomycin, a Ca^2+^-ionophore, did not increase transglutaminase activity, whereas 4-OP increased the activity of the enzyme by 2.5 times.

Not only can ER stress be related to apoptosis induction, but evident cross-talk between ER stress and autophagy has been widely described [43]. Moreover, in accordance with cellular, tissue and microenvironment conditions, autophagy can act as a pro-death or a pro-survival mechanism [44]. It has been observed that 4-NP could simultaneously induce autophagy and apoptosis in Sertoli cells [45] and ovarian granulosa cells [46]. Our data also confirmed these observations in human hepatic cells. Indeed, we provided evidence that 4-OP induced autophagy in HepG2 cells, as demonstrated by the biochemical analyses of two relevant markers of autophagosome maturation: LC3-II, which accumulated in a dose-dependent manner after 24 h of treatment, and p62, which was degraded in parallel, indicating an increase in the autophagic flux. The observation that the use of Baf A1 caused the further accumulation of LC3-II supported this conclusion.

One of the main mechanisms by which APs induce toxicity is the generation of reactive oxygen species [10,47,48]. The first line of defense against oxidants is composed of two enzymes: SOD and CAT. It has been reported that CAT and SOD expression and activity decrease in the presence of 4-OP in the amphibian liver [49], in the rat liver [47] and in the rat kidney [50], whereas they increase in a fish gonadal cell line [51] and in primary liver fish cells [52]. It appears clear that perturbations of the antioxidant system following exposure to APs could be variable and dependent on biological models and cell types in the study. Regarding the antioxidant response, the ADMET prediction suggest that 4-OP has a low probability of activating the Nrf/antioxidant signaling pathway. However, in HepG2 cells, we found that 4-OP induced an increase in both CAT activity and expression after 18 h of treatment. We can suppose that cells were responding to an increase in reactive oxygen species by inducing CAT levels to restore homeostasis. This interpretation is coherent with the observation that the HepG2 cell line is particularly resistant to the action of reactive oxygen species because it has high baseline CAT activity [53]. On the other hand, SOD expression clearly decreased at 25 μM and then increased at a higher concentration, suggesting differential sensitivity in the response involving SOD to different doses of this pollutant.

## 4. Materials and Methods

### 4.1. Alkylphenols

The 4-OP employed in this study was the 4-tert-OP (Merck, Milan, Italy) a branched isomer, whereas 4-NP (Merck) was a linear isomer. Both compounds were prepared as stock solutions in dimethyl sulfoxide (DMSO) at concentrations of 100 mM and diluted in a culture medium for cell treatments. For each treatment, the final concentration of DMSO in the culture medium was less than 0.05%. For APs, a dose-range of 6.25–100 μM was generally used. This dose-range was chosen based on values reported for in vitro studies on other human cell models [12,24,26,34,54,55].

### 4.2. Cell Cultures

All cell lines (HepG2, Caco-2, MRC5, HEK 293, HaCat) were obtained from Interlab Cell Line Collection, National Institute for Cancer Research (Genoa, Italy). HepG2 and Caco-2 cells were cultured in Eagle’s Minimum Essential medium (Life Technologies, Milan, Italy) supplemented with 1% (*v*/*v*) non-essential amino acids, 0.2 mM L-glutamine, 50 units/mL penicillin and 50 µg/mL streptomycin and 10% (*v*/*v*) or 20% (*v*/*v*) fetal bovine serum, respectively; MRC5, HEK 293 and HaCat cells were cultured in Dulbecco’s Modified Eagle medium (Life Technologies) supplemented with 10% (*v*/*v*) fetal bovine serum, 0.2 mM L-glutamine, 50 units/mL penicillin and 50 µg/mL streptomycin. Cells were maintained at 37 ° C in a 5% CO_2_ atmosphere and passaged twice a week.

### 4.3. MTT Assay

To evaluate cell viability, an MTT assay was performed. Here, 24 h before treatments, cells were seeded in 96-well plates and then treated with different concentrations of 4-OP, 4-NP or a mixture of them. To detect mitochondrial dehydrogenase activity, MTT was added to the medium (0.5 mg/mL) and incubated at 37 °C for 1 h. Finally, formazan crystals were solubilized in DMSO, and absorbances were measured at 595 nm (and 655 nm to deduct backgrounds) in a microplate reader. Cell viability was directly proportional to registered absorbance and was expressed as relative viability with respect to vehicle-treated cells.

### 4.4. Western Blot

Western blot analyses were performed to detect protein levels and the expression of several proteins after treatments with 4-OP. After treatments, cells were mechanically harvested and lysed in a buffer containing the following: 20 mM Tris-HCl, pH 7.5, 150 mM NaCl, 1 mM EDTA, 1 mM dithiothreitol, 0.1% sodium dodecyl sulfate, 1% Triton X-100, 1 mM orthovanadate and an inhibitor cocktail (all from Merck). The protein mixtures were resolved in a polyacrylamide denaturant gel and then electrotransferred to a polyvinylidene difluoride (PVDF) membrane (Microtech, Naples, Italy) by using the Trans-Blot Turbo Transfer System (Bio-Rad Laboratories, Milan, Italy). After a blocking step with 5% skimmed milk in Tris-buffered saline (TBS), membranes were incubated with the following primary antibodies, diluted 1:1000 in T-TBS-1% milk: mouse anti-p53 (Santa Cruz, CA, USA); mouse anti-GRP78 (Invitrogen, Milan, Italy); rabbit anti-LC3 (Invitrogen); mouse anti-caspase 3 (Invitrogen); mouse anti-p62 (Invitrogen); rabbit anti-SOD (Elabscience, Houston, TX, USA); rabbit anti-CAT (Elabscience). For normalization, a mouse anti-GAPDH (Santa Cruz) diluted 1:4000 in T-TBS-1% milk was used. As secondary antibodies, horseradish peroxidase-conjugated anti-mouse or anti-rabbit antibodies (Bio-Rad Laboratories) were used for 1 h; finally, immunocomplexes were revealed using a chemiluminescence detection kit (Millipore, Milan, Italy) according to the manufacturer’s instructions.

### 4.5. XBP1 Splicing Detection

HepG2 cells were treated for 4 h with 4-OP or THP in six-well plates, and then, RNA was isolated by using the Trizol reagent (Invitrogen), according to manufacturer’s instructions. To obtain cDNA, 1 μg of RNA was retro-transcribed employing the QuantiTect Reverse Transcription Kit (Qiagen, Milan, Italy). Finally, a conventional PCR was performed to amplify the unspliced and the spliced form for XBP1, with the following primers: 5′-CCTGGTTGCTGAAGAGGAGG-3′ and 5′-CCATGGGGAGATGTTCTGGAG-3′, as previously reported [24]. Amplified cDNA was run on a 2.5% agarose gel and stained with ethidium bromide.

### 4.6. Fluorescent Microscopy

#### 4.6.1. BrdU Incorporation Assay

HepG2 cells were seeded on round glass coverslips (diameter 12 mm) and treated with 4-OP for 18 h. Then, BrdU (Invitrogen) was added to the medium (100 μM) and incubated for 90 min. After washing with phosphate-buffered saline (PBS), fixing with 4% paraformaldehyde for 10 min, permeabilizing with 0.2% Triton X-100 for 5 min and treatment with HCl 1.5 N for 8 min, BrdU incorporation was monitored using an anti-BrdU antibody 1:100 (Merck) and a secondary tetramethylrhodamine-conjugated antibody (Thermo Fisher Scientific, Milan, Italy) at 1:100. Cells were also stained for 10 min with Hoechst 33258 (1 mg/mL) in PBS. Coverslips were mounted with Mowiol (Merck; Milan, Italy) and observed with an AxioSkop40 fluorescent microscope (Carl Zeiss MicroImaging, Inc., Jena, Germany). Images were acquired with an Axiocam MRc5 and processed with the Axiovision 4.2 software (Carl Zeiss MicroImaging Inc.). The number of cells that entered into S-phase was expressed as the ratio between the number of cells incorporating BrdU and the total number of cells.

#### 4.6.2. TUNEL Assay

The Fragment End Labelling (FragEL™) DNA Fragmentation Detection Kit (Merck) was used to evaluate apoptosis in HepG2 cells treated with 4-OP, according to the manufacturer-provided protocol. Microscope observation and image acquisition were performed as described above.

#### 4.6.3. LC3 Detection

HepG2 cells seeded on glass coverslips were treated with 4-OP per 24 h and then fixed and permeabilized as described above. Then, coverslips were incubated for 1 h with the anti-LC3 rabbit antibody (1:100, in 1% bovine albumin serum) and then with an anti-rabbit tetramethylrhodamine-conjugated antibody (1:100, in 0.1% bovine serum albumin) (Thermo Fisher Scientific). After washing, coverslips were mounted with Mowiol and observed with an Olympus CKX41 fluorescent microscope (Olympus Italia srl, Segrate, Italy) and processed with the Olympus CKX41 software (cellSens Dimension 1.5).

### 4.7. Catalase Activity Assay

Catalase activity was measured in HepG2 cells exposed for 18 h to 4-OP. A commercial colorimetric kit from Elabscience was employed, and the assay was performed according to the manufacturer’s protocol. Absorbances were measured at 405 nm. Catalase activity was expressed as the relative activity with respect to vehicle-treated cells.

### 4.8. ADMET Prediction

ADMET-related parameters were predicted by using the online freely accessible platform AdmetLab 2.0 [56]. The 4-OP SMILE (Simplified Molecular Input Line Entry System) string was provided to the platform to predict its chemical–physical and toxicological properties. Among available platforms, AdmetLab 2.0 was chosen for its high coverage of the parameters and its high accuracy and precision in the predictions [57].

### 4.9. Statistics

Data were expressed as means ± standard errors (SE). Student’s *t*-test was used to calculate the statistical significance of differences between treatments, and values of *p* < 0.05 were considered statistically significant.

## 5. Conclusions

With the present study, we highlighted differences in sensitivity to the cytotoxic activity of 4-OP depending on the cell type. A combination of 4-OP and 4-NP produced synergistic effects in some cases, indicating at least a partially different mechanism of action. 4-OP-induced toxicity caused increased apoptosis but also the dysregulation of homeostatic cell processes, such as UPR, autophagy and the antioxidant response, as revealed by experimentations using a human hepatic cell line. Our findings support the idea that 4-OP, like other emerging pollutants that are increasingly abundant in the environment, could represent a potential danger to human health.

## Figures and Tables

**Table 1:** Values of calculated IC_50_ (μM) for cells treated for 24 h with different concentrations of 4-OP, 4-NP or a mixture of them. Values have been calculated from at least three experiments in triplicates. Means and SE (in brackets) are reported. * *p* < 0.05 vs. 4-OP and 4NP; ° *p* < 0.05 vs. 4NP; ^§^ *p* < 0.05 vs. 4-OP.

	HepG2	Caco-2	MRC5	HEK 293	HaCat
4-OP	80.07 (4.46)	105.22 (9.50)	70.88 (2.50)	61.65 (0.13)	37.14 (3.72)
4-NP	83.30 (10.20)	93.26 (9.40)	75.79 (8.25)	62.64 (2.73)	77.43 (1.42) ^§^
4-OP + 4-NP	81.85 (7.22)	53.91 (1.12) *	26.15 (3.13) *	63.84 (4.40)	46.70 (7.15) °

**Table 2:** Predicted ADMET scores of 4-OP.

ADMET Property	Parameter	Value	Reference Range and Interpretation
Absorption	Caco-2 permeability	−4.653	Optimal: higher than −5.15 Log unit
	MDCK permeability	1.5 × 10^−5^	Slow permeability: <2 × 10^−6^ cm/s; medium permeability: 2–20 × 10^−6^ cm/s; high passive permeability: >20 × 10^−6^ cm/s
	Pgp inhibitor	0.316	0–0.3: low; 0.3–0.7: medium; 0.7–1.0: high probability of being an inhibitor
	Pgp substrate	0.002	0–0.3: low; 0.3–0.7: medium; 0.7–1.0: high probability of being a substrate
	HIA (human intestinal absorption)	0.191	0–0.3: low; 0.3–0.7: moderate; 0.7–1.0: high absorption
Distribution	PPB (plasma protein binding)	93.74%	<90% low; ≥90%: high protein-bound
	VD (volume distribution)	5.164	Optimal: 0.04–20 L/kg
	BBB (blood–brain barrier) penetration	0.406	0–0.3: low; 0.3–0.7: moderate; 0.7–1.0: high penetration
	Fu (fraction unbound in plasms)	9.304%	Low: <5%; middle: 5~20%; high: >20%
Metabolism	CYP1A2 inhibitor	0.389	0–0.3: low; 0.3–0.7: medium; 0.7–1.0: high probability of being an inhibitor
	CYP1A2 substrate	0.748	0–0.3: low; 0.3–0.7: medium; 0.7–1.0: high probability of being a substrate
	CYP2C19 inhibitor	0.88	0–0.3: low; 0.3–0.7: medium; 0.7–1.0: high probability of being an inhibitor
	CYP2C19 substrate	0.843	0–0.3: low; 0.3–0.7: medium; 0.7–1.0: high probability of being a substrate
	CYP2C9 inhibitor	0.548	0–0.3: low; 0.3–0.7: medium; 0.7–1.0: high probability of being an inhibitor
	CYP2C9 substrate	0.956	0–0.3: low; 0.3–0.7: medium; 0.7–1.0: high probability of being a substrate
	CYP2D6 inhibitor	0.55	0–0.3: low; 0.3–0.7: medium; 0.7–1.0: high probability of being an inhibitor
	CYP2D6 substrate	0.864	0–0.3: low; 0.3–0.7: medium; 0.7–1.0: high probability of being a substrate
Excretion	Clearance	8.285	High: >15 mL/min/kg; moderate: 5–15 mL/min/kg; low: <5 mL/min/kg
Toxicity	H-HT (human hepatotoxicity)	0.029	0–0.3: low; 0.3–0.7: moderate; 0.7–1.0: high human hepatotoxicity
	AMES toxicity (mutagenicity)	0.013	0–0.3: low; 0.3–0.7: moderate; 0.7–1.0: high toxicity
	Skin sensitization	0.718	0–0.3: low; 0.3–0.7: moderate; 0.7–1.0: high skin sensitization
	Carcinogenicity	0.051	0–0.3: low; 0.3–0.7: moderate; 0.7–1.0: high carcinogenicity
	Eye corrosion	0.978	0–0.3: low; 0.3–0.7: moderate; 0.7–1.0: high eye corrosion
	Eye irritation	0.984	0–0.3: low; 0.3–0.7: moderate; 0.7–1.0: high eye irritation
	Respiratory toxicity	0.475	0–0.3: low; 0.3–0.7: moderate; 0.7–1.0: high respiratory toxicity
	NR-AR (activity on nuclear receptor- androgen receptor)	0.006	0–0.3: low; 0.3–0.7: medium; 0.7–1.0: high probability of being AR agonists
	NR-aromatase (inhibition of nuclear-aromatase receptor)	0.031	0–0.3: low; 0.3–0.7: medium; 0.7–1.0: high probability of being aromatase inhibitors
	NR-ER (activity on nuclear receptor-estrogen receptor)	0.89	0–0.3: low; 0.3–0.7: medium; 0.7–1.0: high probability of being ER agonists
	SR-ARE (activity on antioxidant response element)	0.154	0–0.3: low; 0.3–0.7: medium; 0.7–1.0: high activity
	SR-HSE (activity on heat shock response element)	0.365	0–0.3: low; 0.3–0.7: medium; 0.7–1.0: high activity
	SR-MMP (activity on membrane mitochondrial potential)	0.962	0–0.3: low; 0.3–0.7: medium; 0.7–1.0: high activity
	SR-p53 (activity on p53 regulation)	0.184	0–0.3: low; 0.3–0.7: medium; 0.7–1.0: high probability of being p53 activator

## Data Availability

Data not presented in this manuscript are available on request from the corresponding author.

## Abbreviations

4-NP	4-nonylphenol
4-OP	4-octylphenol
ADMET	Absorption, Distribution, Metabolism, Excretion, and Toxicity
APEOs	alkylphenols ethoxylates
APs	alkylphenols
Baf A1	bafilomycin A1
BrdU	bromodeoxyuridine
CAT	catalase
EDCs	endocrine-disrupting chemicals
GRP78	glucose-regulated protein-78
LC3	microtubule-associated protein 1A/1B-light chain 3
MTT	3-(4,5-dimethylthiazol-2-yl)-2,5-diphenyltetrazolium bromide
SOD	superoxide dismutase
THP	thapsigargin
TUNEL	Terminal deoxynucleotidyl transferase dUTP nick end labeling
UPR	unfolded protein response
XBP1	X-box binding protein 1
